# Exploring SARS-CoV-2 and *Plasmodium falciparum* coinfection in human erythrocytes

**DOI:** 10.3389/fimmu.2023.1120298

**Published:** 2023-03-13

**Authors:** Diana López-Farfán, Nerea Irigoyen, Elena Gómez-Díaz

**Affiliations:** ^1^ Instituto de Parasitología y Biomedicina López-Neyra, Consejo Superior de Investigaciones Científicas (IPBLN, CSIC), Granada, Spain; ^2^ Division of Virology, Department of Pathology, University of Cambridge, Cambridge, United Kingdom

**Keywords:** COVID-19, malaria, novel coronavirus, ACE2, CD147, red blood cells

## Abstract

The co-occurrence and the similarities between malaria and COVID-19 diseases raise the question of whether SARS-CoV-2 is capable of infecting red blood cells and, if so, whether these cells represent a competent niche for the virus. In this study, we first tested whether CD147 functions as an alternative receptor of SARS-CoV-2 to infect host cells. Our results show that transient expression of ACE2 but not CD147 in HEK293T allows SARS-CoV-2 pseudoviruses entry and infection. Secondly, using a SARS-CoV-2 wild type virus isolate we tested whether the new coronavirus could bind and enter erythrocytes. Here, we report that 10,94% of red blood cells had SARS-CoV-2 bound to the membrane or inside the cell. Finally, we hypothesized that the presence of the malaria parasite, *Plasmodium falciparum*, could make erythrocytes more vulnerable to SARS-CoV-2 infection due to red blood cell membrane remodelling. However, we found a low coinfection rate (9,13%), suggesting that *P. falciparum* would not facilitate the entry of SARS-CoV-2 virus into malaria-infected erythrocytes. Besides, the presence of SARS-CoV-2 in a *P. falciparum* blood culture did not affect the survival or growth rate of the malaria parasite. Our results are significant because they do not support the role of CD147 in SARS-CoV-2 infection, and indicate, that mature erythrocytes would not be an important reservoir for the virus in our body, although they can be transiently infected.

## 1 Introduction

The emergence of SARS-CoV-2 in late 2019 put the world on alert and research was quickly directed to characterize the mechanisms of entry and infection of this novel coronavirus. First studies published early in the pandemic confirmed that the angiotensin-converting enzyme 2 receptor (ACE2), a known receptor for SARS-CoV (1), was also used by SARS-CoV-2 (2, 3) along with the serine protease TMPRSS2 necessary for processing the viral spike (S) protein. However, further research proposed that different human receptors could facilitate viral invasion of different tissues. These included CD147 or basigin, and neuropillin-1 (4–6).

CD147 is a transmembrane glycoprotein of the immunoglobulin superfamily which is expressed in many cell types, including human erythrocytes. The non-glycosylated protein is 28 kDa but its molecular weight can increase to 42-66 kDa due to different levels of glycosylation. It has been described that CD147 presents two different forms of N-glycosylation, named LG (low glycosylation) and HG (high glycosylation), which are heterogeneously present on different cell types and tissues (7). In the context of COVID-19, it has been proposed that CD147 could act as an alternative route for SARS-CoV-2 entry in different cellular types where the expression of the ACE2 is low (e.g. pulmonary epithelial cells and lymphocytes) (4, 8). However, other studies have not found evidence supporting the role of CD147 as a functional SARS-CoV-2 receptor (9–11). Indeed, no change in susceptibility to SARS-CoV-2 infection was observed when CD147 was removed from the surface of lung epithelial cells (10).

Another line of evidence suggesting that red blood cells might be a competent niche for the novel coronavirus, is the report that human erythroid progenitors can be infected by SARS-CoV-2 *via* ACE2 expression during erythropoiesis (12). This could be related to the increase of erythroid progenitors in circulation, hypoxia, anaemia and coagulopathies observed in COVID-19 patients (12, 13).

Furthermore, *in-silico* studies have also proposed that the SARS-CoV-2 S and ORF3 proteins might have the ability to bind and to degrade haemoglobin (14). In addition, the band3 protein on the surface of red blood cells has also been postulated as a potential entry point for SARS-CoV-2 by biophysical modelling of proteins interaction (15); and it has been suggested that SARS-CoV-2 could form pores on the erythrocytes membrane and invade them *via* the membrane attack complex domain of the S protein (16). All this evidence suggest human erythrocytes could be a competent niche for SARS-CoV-2 *via* the receptor CD147 or by alternative mechanisms. To our knowledge this hypothesis has not been tested yet.

CD147 is also essential for red blood cells invasion by the malaria parasite *Plasmodium falciparum* (17) opening up a possible SARS-CoV-2/malaria co-infection scenario. The intra-erythrocytic cycle of *P. falciparum* takes around 48h to complete and involves several stages. It begins when the invasive form of the parasite, the merozoite, invades a new erythrocyte. In the first 12 hours post-invasion, the parasite develops into the ring form; and at 18 h, the trophozoite. Approximately 36h post invasion, the mitosis occurs and the parasite cell progresses to a multinucleated stage named schizonts which contains new merozoites that are released to the bloodstream to start a new cycle (18). We and others have already reported co-infection cases in malaria endemic countries (19, 20). Apart of this co-occurrence, some computational studies have found immunodominant epitopes shared by SARS-CoV-2 and *P. falciparum* proteins (21). *P. falciparum* is also known to remodel the membrane of their erythrocyte host (22, 23) and this could potentially enhance viral invasion by SARS-CoV-2. Experimental studies investigating this hypothesis are missing.

In this work, we tested whether mature erythrocytes could be bound or invaded by SARS-CoV-2, and therefore, serve as a niche or reservoir for the virus. We first carried out *in vitro* infection experiments with SARS-CoV-2 pseudoparticles in HEK 293T cells transiently expressing the CD147 or the ACE2 receptors. We found that transient expression of ACE2, but not CD147, allowed entry and infection by SARS-CoV-2 pseudoviruses. Although we did not find evidence that CD147 could act as a functional receptor for SARS-CoV-2, we decided to explore the hypothesis of an alternative mechanism of adhesion and/or entry to human erythrocytes using a SARS-CoV-2 wild type virus isolate. We reported adhesion and entry of SARS-CoV-2 to red blood cells, although at low frequency. We also tested if the presence of *P. falciparum* could make erythrocytes more vulnerable to SARS-CoV-2 infection. We found that *P. falciparum* did not facilitate the entry of SARS-CoV-2 into erythrocytes, and these cells were not frequently co-infected.

## 2 Methods

### 2.1 Cell culture

HEK 293T cells were cultured in Dulbecco’s modified Eagle’s medium (DMEM) with high glucose (Sigma D6429) supplemented with 5% heat-inactivated foetal bovine serum (iFBS) and 1x penicillin-streptomycin (P/S; Sigma P4333). VeroE6 cells were cultured in DMEM (Sigma D6429) supplemented with 10 mM HEPES, 1x MEM non-essential amino acid solution (Sigma M7145), 10% iFBS and 1x P/S.

### 2.2 Parasite culture

*Plasmodium falciparum* blood stage cultures (3D7) were maintained in RPMI 1640 (Gibco, ref. 51800-035) supplemented with 25 mM HEPES, 2.1 g/mL NaHCO_3_, 5% AlbuMAX II (Gibco), 0.37 mM hypoxhantine and 5% human erythrocytes. Cultures were incubated at 37°C under 1% CO_2_, 3% O_2_ and 96% N_2_ gas mixture. Parasite development was monitored by Giemsa staining, and cultures were maintained as previously described (24).

### 2.3 Human erythrocytes

Human whole blood of healthy donors was obtained from the Andalusian Public Health System Biobank. Blood was centrifuged at 2,000 rpm for 10 min, plasma and buffy coat were removed and the red blood cell pellet was washed three times in RPMI 1640 supplemented with 25 mM HEPES. Finally, the packed red blood cell pellet was resuspended with an equal volume of complete RPMI 1640 medium (cRPMI), this 50% haematocrit medium was stored at 4°C and used within three weeks for parasite culture and SARS-CoV-2 infection experiments.

### 2.4 Plasmids and lentiviral pseudovirus generation

pNL4-3.Luc.R-E- (NIH-AIDS reagent program, catalog n° 3418) is an HIV-1-luciferase reporter vector. It contains the luciferase gene flanked by the long-terminal regions (LTRs) for integration into the host genome, gag, pol, tat and rev genes. However, it is defective for nef, env and vpr genes, only allowing a single round of replication. To produce infectious pseudovirions, it needs to be co-transfected with a plasmid expressing an envelope protein: the vesicular stomatitis virus glycoprotein (VSV-G) envelope expressing plasmid (pMD2 VSV-G, Addgene, catalog n° 12259) as a positive control; or the pcDNA3.1 SARS-CoV-2 S prot-HA, a plasmid expressing the codon-optimized SARS-CoV-2 spike protein from isolate Wuhan-Hu-1 with the HA-tag. An empty pcDNA3.1(+) (Invitrogen) plasmid was used as a negative control.

Pseudovirus were produced by co-transfection of HEK 293T cells (plated in 6-well plates) with pNL4-3.Luc.R-E- (800 ng) and an envelope plasmid (800 ng): pMD2 VSV-G, pcDNA3.1 SARS-CoV-2 S prot-HA or pcDNA3.1(+) for no-envelope control. Transfection mixtures were made using 7.5 µL of TransIT^®^-LT1 Transfection Reagent (Mirus) and 250 µL of Opti-MEM I media (Gibco). Mock transfection was performed without plasmids as an additional control. Medium was changed at 24h and pseudovirus-containing supernatants were harvested at 48 h post-transfection, centrifuged at 500 x g for 5 min to remove cellular debris, aliquoted and used immediately or stored at −80°C for later use.

### 2.5 Reverse transfection of ACE2 or CD147 and TMPRSS2 into HEK293T cells

Transient expression of the human receptors ACE2 or CD147 and the protease TMPRSS2 was performed by reverse transfection the plasmids pcDNA3.1-ACE2, pcDNA3.1-CD147 and pcDNA3.1-TMPRSS2 into HEK 293T cells. 24 hours prior to transfection, cells were plated in a T75 flask to be 70-80% confluent the following day. Next day, cells were trypsinized, counted and resuspended at a density of 1.6 x 10^5^ cells/mL. The transfection master mix was prepared with 5 µg of the plasmid of interest, 45 µL of TransIT^®^-LT1 Transfection Reagent (Mirus) and 1.5 mL of Opti-MEM media (Gibco), the mixture was incubated 15 min and then 11.04 mL of cell suspension was added to the mix. Cells were plated in quadruplicate in a 96-well plate (100 µL/well) and incubated for 24h.

### 2.6 Luciferase assay

96-well plates seeded with HEK 293T transfected with the receptors or mock transfected (control cells) were infected with 100 µl of pseudovirus-containing supernatants, plates were incubated for 48h and analysed for luciferase activity using the Luciferase Assay System (E1500, Promega), following the manufacturer’s protocol. Cells were rinsed once in 1x PBS and lysed with 40 μL of lysis reagent per well, 20 μL of cell lysate was transferred to a white 96-well plate and 50 µL of Luciferase Assay reagent was added to each well, luminescence was measured in a TECAN infinite 200 instrument with 7.5 sec of readout time.

### 2.7 Western blot

HEK 293T cells transfected with 2.5, 3.5 and 5 µg of pcDNA3.1-CD147 were lysed 24h and 48h post-transfection with 1x RIPA buffer (abcam) and 1x Complete EDTA free protease inhibitor cocktail (Roche). Cell lysates were quantified by DC protein Assay (Bio-Rad) following manufacturer’s instructions and then mixed with 4x Laemmli’s sample buffer (Bio-Rad) with 10% β-mercaptoethanol. Protein extracts were loaded and run on 10% SDS-PAGE gels and transferred onto a PVDF membrane (Bio-Rad). Membranes were blocked with 5% skimmed milk/TBST 1X (TBS1X-0.1% Tween 20) for 1 h and then incubated overnight at 4°C with primary antibodies; rabbit anti-CD147 (abcam ab108308; 1:1,000 dilution) and mouse anti-GAPDH (Affinity Biosciences, T0004; 1:10,000 dilution) in blocking solution. Following washing, membranes were incubated with the respective secondary antibodies for 1 h at room temperature; goat anti-rabbit IgG (IRDye^®^ 800CW) (ab216773) and goat anti-mouse IgG (IRDye^®^ 680RD) (ab216776) diluted 1:20,000. After washing, the fluorescence signal was visualized using an Odyssey CLx Imaging System (LI-COR Biosciences).

### 2.8 SARS-CoV-2 virus culture and infection assays

SARS-CoV-2/NL/2020 strain passage 3 was obtained from the European Virus Archive GLOBAL (EVA). Viral titre was determined as follows by plaque assay (protocol kindly shared by C. Suñé). Confluent monolayers of VeroE6 cells were grown on 6-well plates and incubated with 300 µL of a 10-fold serial dilution of the virus stock in DMEM supplemented with 0.2% BSA, 10 mM HEPES and 1x MEM, for 1 h at 37°C. After the adsorption hour, cells were overlaid with MEM 1x supplemented with 10% FSB, 2 mM Glutamine, 1x penicillin-streptomycin, plus 0.1% agarose and incubated for 72h at 37°C. Next, cells were fixed with 1% glutaraldehyde for 20 min. Medium was removed by aspiration and cells were washed with distilled water. Lysis plaques were stained with 0.1% crystal violet for 30 min at room temperature and washed with distilled water. Plaques were counted and quantified as plaque-forming units (PFU) per mL. To generate viral stocks, SARS-CoV-2 was propagated on VeroE6 cells in DMEM supplemented with 2% FSB, at MOI of 0.01. After 72h the virus-containing supernatant was collected, centrifuged for 10 min at 2.000 rpm, aliquoted and titrated as described above. Aliquots were stored at −80°C for later use.

Erythrocytes, stored in cRPMI medium at 50% haematocrit (see above), were quantified in a Neubauer chamber. 2 x 10^6^ erythrocytes were incubated in protein low bind tubes with SARS-CoV-2 viral supernatant at a MOI 2, for 1h at 37°C with gentle agitation every 15 min to maintain the erythrocytes in suspension. After centrifugation at 1,500 rpm for 3 min, the supernatant was discarded and the erythrocytes were washed four times with 0.5 mL of phenol free RPMI to remove unbound virus. Erythrocytes were then resuspended at 5 x 10^4^ cells/µL and 500,000 cells were seeded on microscope round cover glasses of 12 mm (VWR) previously treated with 50 uL Concanavalin A (Sigma, 5 mg/mL in water) for 20 min at 37°C in 24-well plates. Erythrocytes were allowed to settle for 20 min at 37°C and unbound cells were rinsed with two PBS washes. Seeded cells were fixed with 4% paraformaldehyde (PFA)/0.0075 glutaraldehyde (GA) in PBS for 20 min at 37°C and analysed by indirect immunofluorescence microscopy.

Erythrocytes from a *P. falciparum* culture were quantified in a Neubauer chamber and the parasitemia was determined by Giemsa-stained blood smears. 2 x 10^6^ erythrocytes from a *P. falciparum* culture at 5.5% parasitaemia were incubated in protein low bind tubes with SARS-CoV-2 at a MOI 2 for 1h at 37°C with gentle agitation every 15 min to maintain cells in suspension. After incubation, erythrocytes were washed, seeded, and fixed on round cover glasses, as described before. Indirect immunofluorescence was performed using an antibody against the SARS-CoV-2 N protein and DAPI staining for detection of the parasite DNA.

*P. falciparum* culture at 7% parasitaemia was incubated with SARS-CoV-2 at a MOI 2 for 1h at 37°C in a T-25 flask. A control culture was incubated with DMEM, 2% FSB. After incubation, erythrocytes were centrifuged at 1,500 rpm for 5 min, the red blood cell pellet was resuspended in 500 µL of cRPMI and transferred to a protein low-binding Eppendorf. After centrifugation, erythrocytes were resuspended in 50 µL of cRPMI and diluted with 50 µL of cRPMI medium, 5% haematocrit (to dilute the culture below 3.5% parasitemia), the cell suspension was transferred to a well in a 96-well plate and incubated for 24 and 48 hours at 37°C. Culture development was monitored by Giemsa-stained blood smears and quantified by microscopic examination.

### 2.9 Immunofluorescence microscopy

Coverslips with fixed erythrocytes were rinsed with PBS twice and permeabilized with 0.1% Triton^®^ X-100 in PBS for 15 min, after three PBS washes, free aldehyde groups were quenched with 30mM glycine for 5 min followed by PBS washes. Then, cells were blocked with 3% BSA/1x PBS for 45 min. The primary antibody, mouse anti-SARS-CoV-2 N protein (abcam, ab281300), was diluted 1:1,000 in antibody solution (1% BSA/0.5% Tween^®^ 20/1x PBS) and centrifuged at 13,300 rpm for 1 min to remove aggregates prior to incubation for 2h at room temperature on a horizontal shaker. After three washes with PBS for 10 min, the secondary antibody goat anti-mouse Alexa Fluor 594 (Invitrogen, A11020), was diluted 1:500 and incubated for 45 min. After three washes with PBS for 10 min, the coverslips were mounted on glass slides using VECTASHIELD^®^ Vibrance™ mounting medium (Vector laboratories). Images were acquired using a fluorescence microscope (DMi8, Leica, Germany), with 100x objective and the LAS X software (Leica). Erythrocytes were visualized by phase contrast (gray). Stacks (0.19-μm z step) acquisition was performed to capture 3D images of the red blood cells. Maximum intensity projections of anti-N and DAPI channels were performed using ImageJ 1.52p software (National Institutes of Health). Quantification of SARS-CoV-2 viral particles bound to the erythrocytes after four washes was performed in maximum intensity projection images. The percentage of erythrocytes with viral particles was calculated counting 2,490 red blood cells and 3,418 red blood cells in the case of the *P. falciparum* cultures.

### 2.10 Biosafety statement

All work performed was approved by the local genetic manipulation (GM) safety committee. All work with live SARS-CoV-2 was performed in biosafety laboratory level 3 facilities at the IPBLN, Granada, Spain.

## 3 Results

### 3.1 Overexpression of the CD147 receptor in HEK 293T cells does not mediate the infection of SARS-CoV-2 pseudoparticles

**Figure 1:**
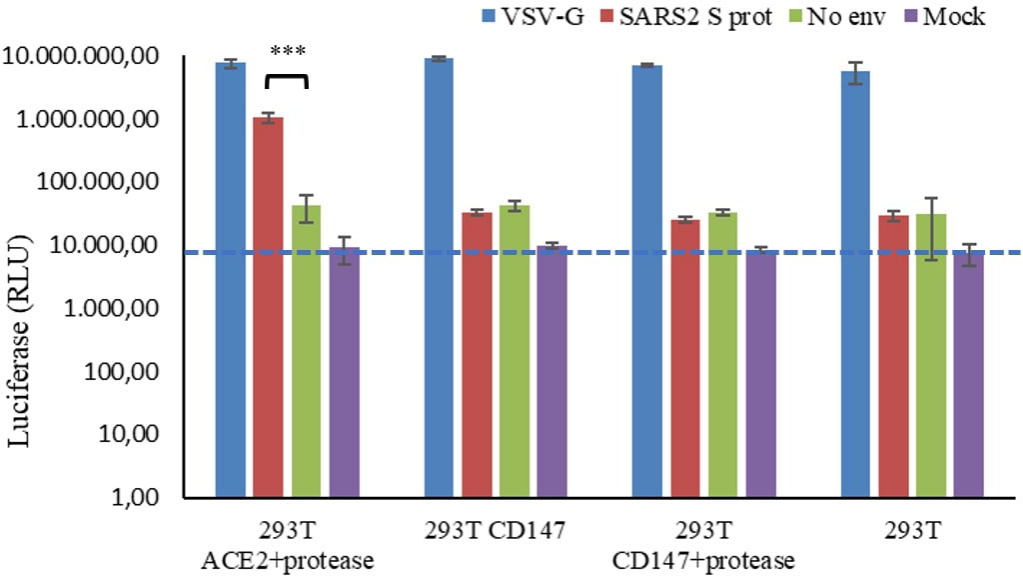
Luciferase activity of SARS-CoV-2 pseudovirus infection in HEK 293T cells expressing the ACE2 o the CD147 receptors. HEK 293T cells were transiently transfected with ACE2 and the protease TMPRSS2, or with CD147 alone or in combination with the TMPRSS2 protease. At 24 h post-transfection, cells were infected with SARS-CoV-2 (SARS2 S prot), VSV-G or naked (No Env) pseudovirions. Mock indicate fresh culture medium. After 48 h, cells were lysed and luciferase activity was measured. The average of three independent experiments conducted with quadruplicate samples are shown. Student’s t-test was performed, ***p<0.001. The blue line indicates background signal of mock infection (no virus). RLU: relative luminescence units in log scale.

Due to contradictory evidence of the function of CD147 as a truly SARS-CoV-2 receptor (9–11), we first investigated whether the transient overexpression of CD147 in HEK 293T cells could mediate the internalization of SARS-CoV-2 pseudovirions. In order to do this, luciferase reporter pseudovirions based in lentiviral vectors were constructed (25) where the cellular entry will be mediated by the expression of the SARS-CoV-2 spike (S) protein. In the same system, the vesicular stomatitis virus glycoprotein (VSV-G) was used as a positive control of infection due to its broad cell-type tropism. In addition, particles without the S protein were generated as a negative control. Infection was measured by a luciferase activity assay at 48 hours post-infection (h p.i.) in HEK 293T cells expressing the ACE2 or the CD147 receptors. The amount of luciferase activity reflects the expression of integrated proviruses (26). We found that only cells expressing the ACE2 receptor and the TMPRSS2 protease were permissive for the entry of SARS-CoV-2 pseudovirions (**Figure 1**; **Supplementary Figure S1**). As a positive control, assays were performed with the same amount of VSG-G pseudoviral supernatant, this showed higher luciferase activity in all the assays. In addition, pseudoparticles without an envelope protein were producing a similar level of luciferase activity as the mock supernatant without pseudoparticles.

**Figure 2:**
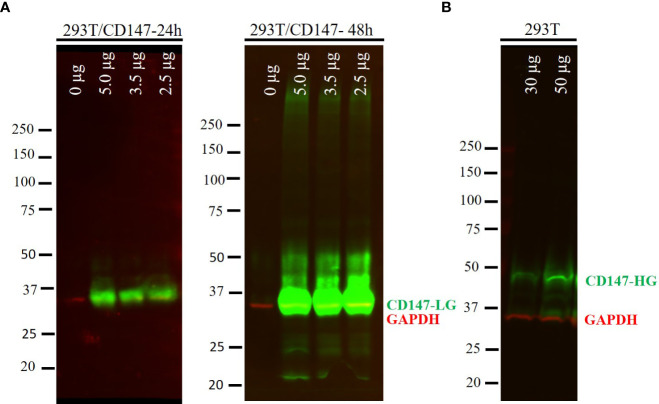
Protein expression of CD147 in HEK 293T cells. **(A)** HEK 293T cells transfected with different amounts of a plasmid expressing the CD147 protein (0, 2.5, 3.5 and 5.0 µg). Whole cell lysates were harvested at 24 and 48 h post-transfection and analysed by Western blotting. Proteins were loaded at 25 µg per line. **(B)** Endogenous expression of CD147 in HEK 293T cells was detected loading 30 and 50 µg of the whole cell extract. Low glycosylated CD147 (CD147-LG, ~32 kDa), high glycosylated CD147 (CD147-HG, ~42 kDa). GAPDH (34 kDa) was used as a loading control. Overlay of the simultaneous detection of CD147 (800 nm channel-green) and GAPDH (700 nm channel-red) fluorescent signals.

As SARS-CoV-2 pseudoparticles were not internalized in the CD147-transfected cells, we decided to test whether CD147 was properly expressed. The expression of CD147 in HEK 293T was confirmed by Western blot analysis (**Figure 2**). As observed in **Figure 2A**, when CD147 was transiently overexpressed, most of the protein was in the low glycosylated form (LG-CD147, ~32 kDa), and the amount increased with time. We also analysed the endogenous expression of CD147 in HEK 293T and we found that this cell line expressed low but detectable levels of LG-CD147 and high glycosylated CD147 (HG-CD147, ~42 kDa) (**Figure 2B**). Despite the expression of the two CD147 isoforms, we found not significant luciferase activity for SARS-CoV-2 pseudovirions when using non-transfected HEK 293T cells (**Figure 1**). In addition, the transient expression of ACE2 in HEK-293T was also analysed by immunofluorescence and flow cytometry, we found heterogeneous levels of expression in 10% of the transfected population (**Supplementary Figure S1**). Although the percentage of ACE2 expression was not high, it was enough to detect a 24.6-fold increase of SARS-CoV-2 pseudovirus entry in HEK 293T cells previously transfected with ACE2 and TMPRSS2 compared to the negative control (No env) in luciferase assays (**Figure 1**).

### 3.2 Low entry of SARS-CoV-2 to human erythrocytes *in vitro*

**Figure 3:**
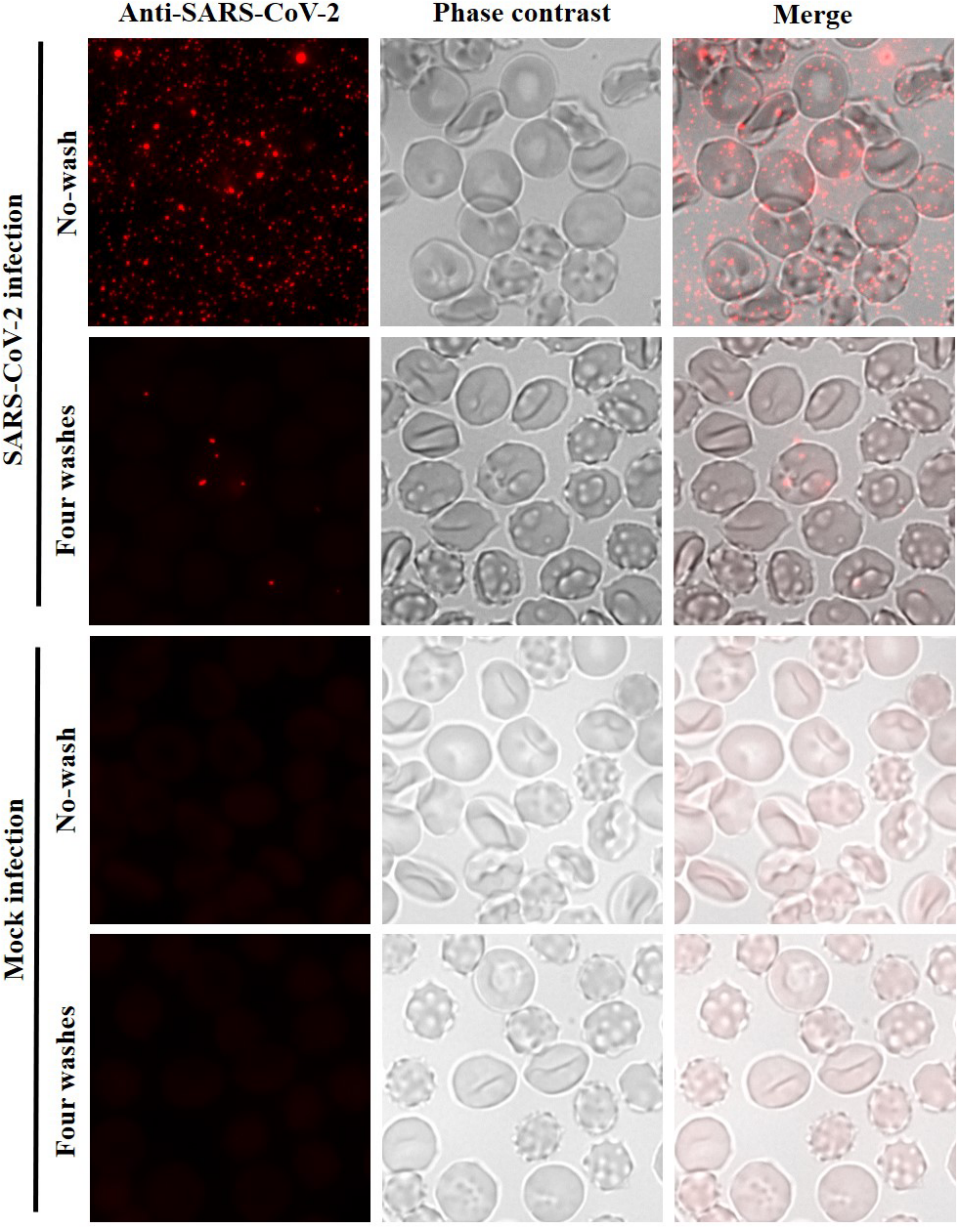
Low entry of SARS-CoV-2 to human erythrocytes. Immunofluorescence analysis using an anti-SARS-CoV-2 nucleocapsid antibody for detection of SARS-CoV-2 in infection assays with human erythrocytes. Erythrocytes were incubated with SARS-CoV-2 at MOI of 2 or with infection medium without virus (mock infection). No washed and four washed samples were analysed. Erythrocytes were visualized by phase contrast (gray). Maximum intensity projections of three-dimensional stacks are shown for the red channel. The percentage of erythrocytes with viral particles (red dots) attached after four washes was calculated counting several fields with a total of 2,490 red blood cells (10.94%; SD 1.56%).

Since CD147 alone does not appear to be a functional receptor for SARS-CoV-2 infection, we explored whether SARS-CoV-2 was able to adhere and/or enter to human erythrocytes *via* an alternative mechanism. Infection assays were performed with the SARS-CoV-2/NL/2020 strain. Erythrocytes were incubated for 1 h with SARS-CoV-2 at a multiplicity of infection (MOI) of 2. After four washes with phenol red-free RPMI to remove unbound virus, erythrocytes were seeded on microscope coverslips, fixed and analysed by indirect immunofluorescence microscopy (IF) using an antibody against the SARS-CoV-2 nucleocapsid (N) protein. Adhesion and entry of SARS-CoV-2 to red blood cells could be observed (**Figure 3**) but to a lower rate in comparison with VeroE6 infected under the same conditions (**Supplementary Figure S2**). After four washes, 10.94% of the erythrocytes (standard deviation, SD 1.56%) had viral particles attached. However, virions were not accumulated inside the erythrocytes or the plasma membrane as observed in VeroE6 cells (**Supplementary Figure S2**).

### 3.3 *P. falciparum* does not promote the entry of SARS-CoV-2 to erythrocytes

**Figure 4:**
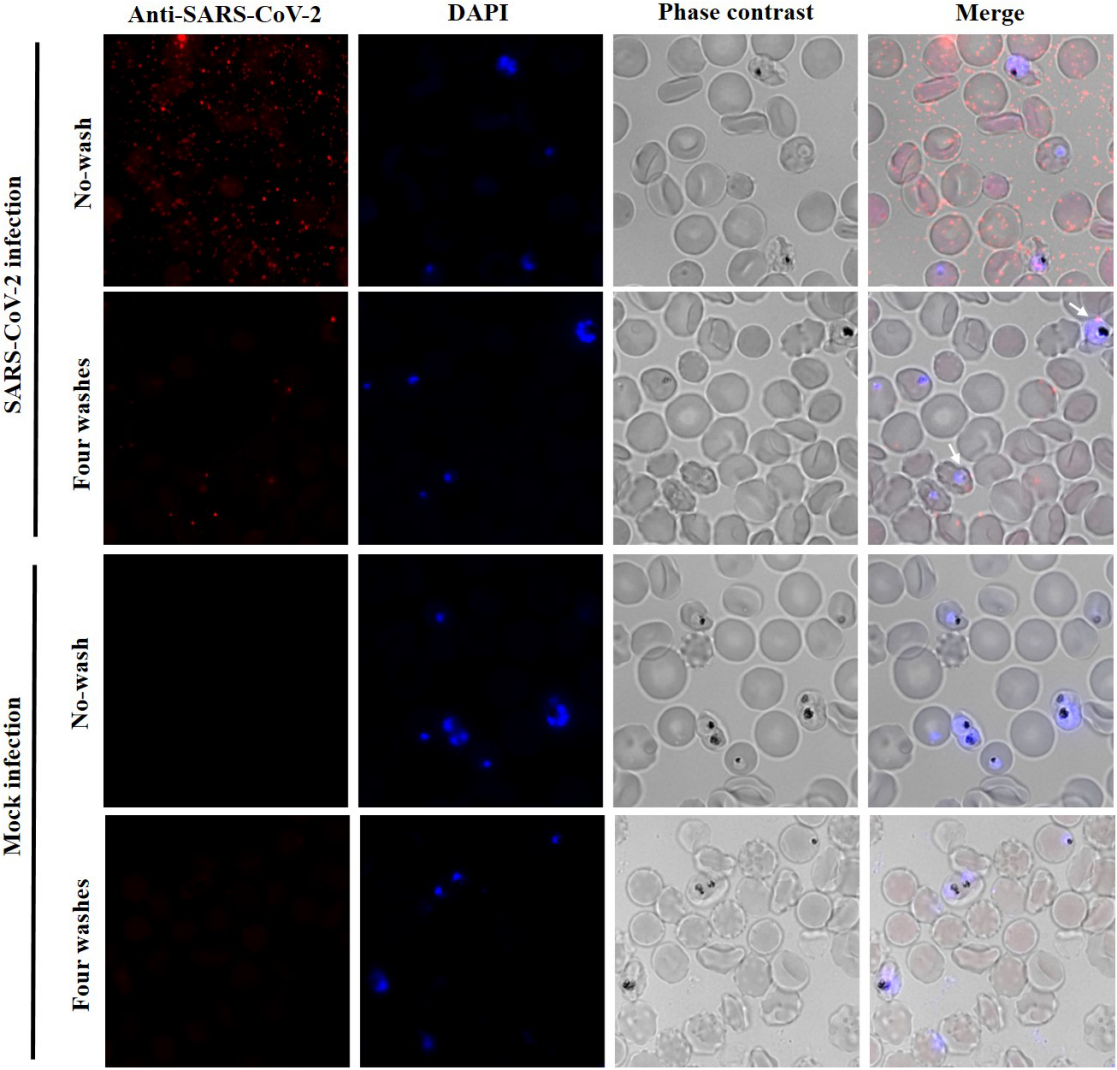
Low adhesion or entry of SARS-CoV-2 to *P. falciparum* infected erythrocytes. Immunofluorescence analysis with an anti-SARS-CoV-2 nucleocapsid antibody. *P. falciparum* culture at 5.5% parasitaemia was incubated with SARS-CoV-2 at MOI of 2 or with infection medium without virus (mock infection). No washed and four washed samples were analysed. *P. falciparum* DNA was stained with DAPI. Erythrocytes were visualized by phase contrast (gray). Maximum intensity projections of three-dimensional stacks are shown for red and DAPI channels. The percentage of *P. falciparum* infected erythrocytes with viral particles (red dots) attached after four washes was calculated counting several fields with a total of 3,418 red blood cells (9.13%; SD 1.82%).

As *P. falciparum* remodels the erythrocyte membrane (22, 23), this could render malaria-infected red blood cells (iRBCs) more vulnerable to the adhesion and entry of SARS-CoV-2, potentially, promoting co-infection. To test this hypothesis, a *P. falciparum* culture at 5.5% parasitaemia was incubated for 1 h with SARS-CoV-2 at MOI:2. After incubation, erythrocytes were washed with phenol red-free RPMI, seeded on coverslips, fixed and analysed by IF using a SARS-CoV-2 N antibody for SARS-CoV-2 detection, and DAPI staining for detection of the parasite DNA. As observed in **Figure 4**, after four washes, few viral particles were detected (1 to 5 particles per cell) in a low percentage of the parasitized red blood cells (9.13%; SD 1.82%). In addition, the potential co-infection of SARS-CoV-2 and *P. falciparum* in red blood cells was mainly observed during the ring stage of the parasite. These results suggest that the presence of *P. falciparum* inside erythrocytes does not facilitate or increase the SARS-CoV-2 entry to these cells.

### 3.4 SARS-CoV-2 does not impact red blood cell development of *P. falciparum*

We also wanted to test whether the presence of SARS-CoV-2 in malaria-infected erythrocytes could affect the parasite infection process, especially invasion and growth. To test this, we incubated a *P. falciparum* culture at high parasitaemia with SARS-CoV-2 for 1 h at MOI:2. After incubation, culture cells were washed once with RPMI and further incubated for 24 and 48 h at 37°C in a 96-well plate. After incubation, culture development was monitored by Giemsa-stained blood smears that were quantified by microscopic examination. We found no significant effect in *P. falciparum* parasitaemia and cell stages between SARS-CoV-2 pre-incubated cultures and control cultures incubated with infection medium without the virus (**Supplementary Table S1**).

## 4 Discussion

The CD147 receptor has been proposed as an alternative receptor for SARS-CoV-2 in cellular types where the ACE2 expression is low (4). However, recent studies have not found enough evidence supporting this idea (9–11). In agreement with this, in this study we report that transient expression of CD147 in HEK 293T cells does not result in SARS-CoV-2 pseudoviruses entry.

We thought that the contradictory results regarding CD147-mediated entry could be due to heterogeneous glycosylation of the expressed protein in different cell lines or tissues analysed (7). In our study, we have confirmed that HEK 293T cells express, although to a lower extent, detectable endogenous levels of two isoforms of CD147: the high glycosylated HG-CD147 form (~42 kDa) and a low glycosylated LG-CD147 form (~32 kDa). It has been reported that different cell types express HG/LG CD147 to different ratios (27). However, we found that neither the endogenous expression nor the overexpression of LG-CD147 in HEK 293T cells was enough to mediate SARS-CoV-2 pseudovirus entry (**Figure 1** **).** Indeed, it has been confirmed that HEK 293T are not permissive cells for SARS-CoV-2 infection (28).

Some studies have reported that only fully glycosylated CD147 can be found on plasma membrane and be biologically functional, while other studies have reported that both isoforms can be detected on the plasma membrane (27). It is important to indicate that the overexpression of two highly glycosylated isoforms of recombinant CD147 did not show direct interaction with the S protein (10).

Apart from CD147, alternative mechanisms for SARS-CoV-2 entry to human erythrocytes have been proposed based on computational analysis of epitopes shared by SARS-CoV-2 and *P. falciparum* proteins (14, 16, 21).

Here, we experimentally tested the hypothesis of a possible scenario of infection of human erythrocytes with SARS-CoV-2. Our immunofluorescence analysis showed that SARS-CoV-2 could adhere and enter to erythrocytes but less efficiently compared to a SARS-CoV-2 permissive cell line such as VeroE6. Viral particles did not accumulate inside red blood cells and only 1-5 viral particles were observed in a low percentage of cells (10.9% of erythrocytes) (**Figure 3**).

Due to erythrocyte membrane remodelling after *P. falciparum* infection (23), we also investigated whether malaria-infected red blood cells could be more permissive to viral infection. This could lead to a co-infection scenario by the two pathogens in human red blood cells. However, the incubation of a *P. falciparum* culture with SARS-CoV-2 showed similar results that incubation with uninfected erythrocytes, and only 9.1% of the parasitized erythrocytes showed viral particles attached (**Figure 4**), indicating that the interaction between the two pathogens is not frequent.

Our results suggest that mature erythrocytes in the bloodstream are not a critical niche for SARS-CoV-2 that could contribute to the spreading or hiding of the virus. This agrees with previous studies that report low frequency detection and quantity of viral RNA in the blood from COVID-19 patients (29, 30). In addition, PCR-positive plasma was unable to be cultured in cells. This indicates no presence of infective virions in blood from COVID-19 patients or donors (30, 31).

A possible limitation of this study is that experiments with red blood cells were carried out using 2D *in vitro* cultures, with blood obtained from healthy donors and not with COVID-19 patients. This could be important since damage and clumps of red blood cells in blood vessels have been reported in COVID-19 patient biopsies (32, 33). In addition, complement activation products and S protein binding have been detected in the erythrocyte membrane COVID-19 patients (34). Other possible limitations could be the MOI used and the viral incubation time. Conditions used here were on the high range for the detection of adherence and entry of SARS-CoV-2 to VERO E6, according to previous studies, but there is the possibility that these parameters might not be optimal for erythrocytes.

Our *in vitro* study complements previous field studies reporting non-negligible but low frequency of SARS-CoV-2 and *P. falciparum* co-infection in malaria endemic countries in Africa (19, 20). In these studies, the low co-occurrence of COVID-19 and malaria was in part attributed to cross-immunity due to common immunodominant epitopes shared between *P. falciparum* and SARS-CoV-2 (21, 35–37). Possible mechanisms that would explain the low rate of viral entry to the erythrocytes could be the involvement of secondary SARS-CoV-2 receptors such as neurophilin-1 (5, 6) or the band3 protein, as it has been previously postulated (15).

In this contribution, we designed a novel pseudovirus system that allowed us to show the no involvement of the CD147 receptor in SARS-CoV-2 infection. Secondly, we settled a novel culture approach infecting human red blood cells with a wild type SARS-CoV-2 that allowed us to test a possible co-infection scenario between this novel coronavirus and the human malaria parasite *P. falciparum*. To our knowledge, this is the first study that has experimentally tested the hypotheses of SARS-CoV-2 erythrocyte invasion and co-infection with the malaria parasite. Our results contribute to a better understanding of the SARS-CoV-2 potential cellular niches, and suggest that the damage to the red blood cells, blood vessels, and other coagulopathies observed in severe COVID-19 patients (32, 38) would not be due to viral infection of red blood cells.

## Data availability statement

The original contributions presented in the study are included in the article/**Supplementary Material**. Further inquiries can be directed to the corresponding authors.

## Author contributions

EG-D and NI conceived and designed the original study. DL-F performed the experiments and wrote the manuscript. EG-D and NI supervised the project, edited and reviewed the manuscript. All authors contributed to the article and approved the submitted version.
