# Supplementary material for: Exploring SARS-CoV-2 and Plasmodium falciparum coinfection in human erythrocytes
Source: Front Immunol. 2023 Mar 13;14:1120298. doi: 10.3389/fimmu.2023.1120298 (PMC10041564; doi:10.3389/fimmu.2023.1120298)
Supplement: Supplementary file 1 [file DataSheet_1.pdf]

Supplementary Material

Supplementary Figures and Tables

A.

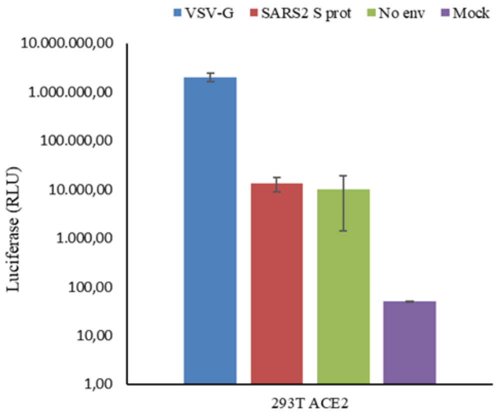

B.

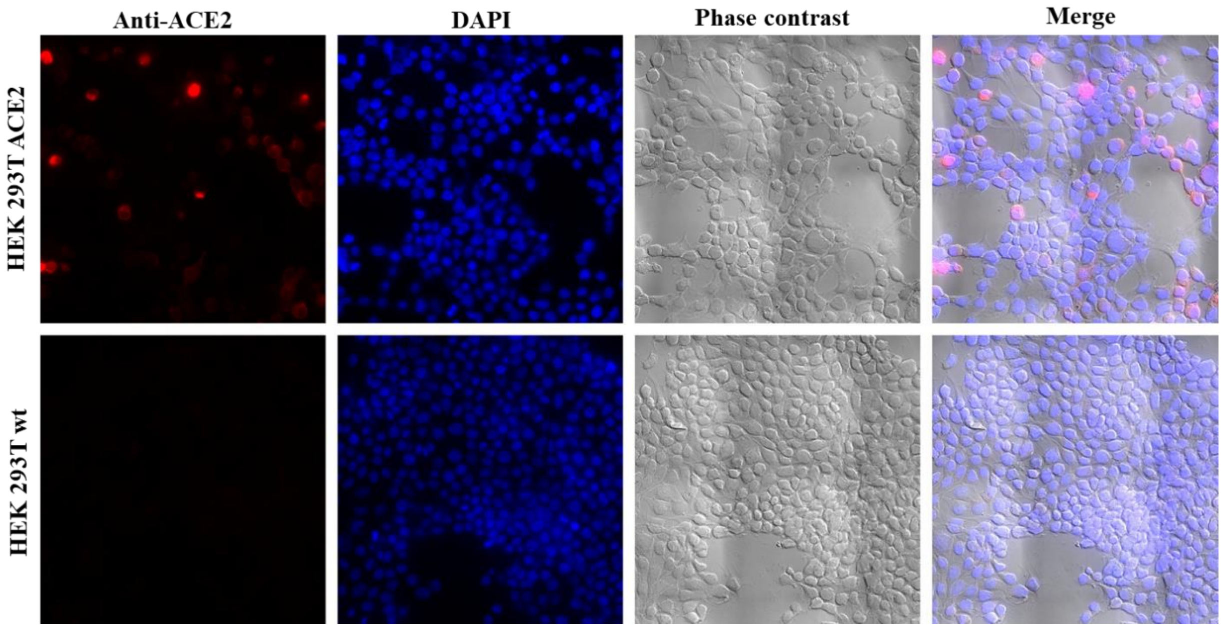

C.

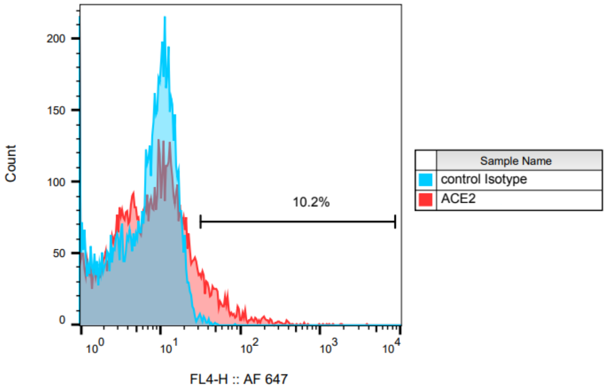

Supplementary Figure S1. (A) Luciferase activity of SARS-CoV-2 pseudovirus infection in HEK 293T cells expressing the ACE2 receptor. HEK 293T cells transiently transfected with ACE2 were infected with SARS-CoV-2 (SARS2 S prot), VSV-G or naked (No Env) pseudovirions. Luciferase activity was measured after 48 h. The average of two experiments conducted with quadruplicate samples is shown. RLU: relative luminescence units in log scale. **(B)** Analysis of ACE2 expression in HEK 293T transfected cells after 48 h of incubation. Immunofluorescence with an anti-ACE2 antibody (R&D Systems, AF933; 15 µg/mL) and an anti-goat IgG Alexa fluor 647 (ab150131) as secondary antibody. **(C)** FACS analysis of ACE2 transient expression in HEK 293T. Fluorescence distribution of ACE2 expressing cells stained with Alexa fluor 647. Normal goat IgG control (R&D Systems, AB-108-C) was used as control isotype.

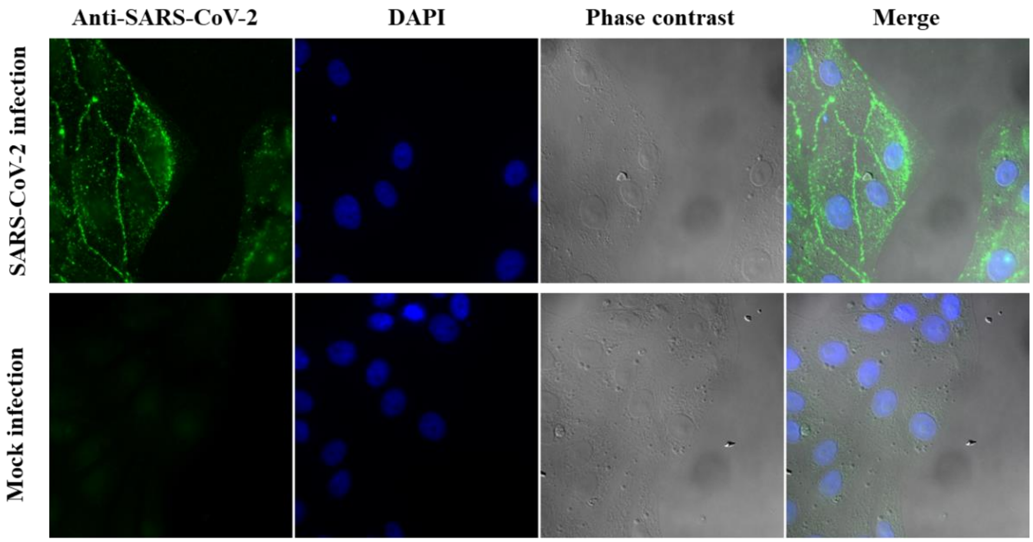

Supplementary Figure S2. SARS-CoV-2 infection in VeroE6 cells. Immunofluorescence analysis with an anti-SARS-CoV-2 nucleocapsid antibody. VeroE6 cell cultures were incubated with SARS-CoV-2 at MOI of 2 or with infection medium without virus (mock infection) for 1 h. Four washed samples are shown.

Supplementary table S1. *P. falciparum* culture development after SARS-CoV-2 exposure. Parasite cell stages correspond to rings (r), trophozoites (t), schizonts (s).

***P. falciparum* culture**	**Incubation time after SARS-CoV-2 exposure**	**Total parasitaemia (cell stages percentage)**
SARS-CoV-2 pre-incubated	24h	3.87% (2.06% r, 1.81% s)
Control		4.02% (2.68% r , 0.73% s, 0.6% t)
SARS-CoV-2 pre-incubated	48h	6.22% (1.18% r, 3.7% s, 1.34 t)
Control		5.57% (1.53% r, 3,07% s, 0,96% t)
